# Just-in-Time Information Improved Decision-Making in Primary Care: A Randomized Controlled Trial

**DOI:** 10.1371/journal.pone.0003785

**Published:** 2008-11-21

**Authors:** Jessie McGowan, William Hogg, Craig Campbell, Margo Rowan

**Affiliations:** 1 Faculty of Family Medicine, University of Ottawa, Ottawa, Canada; 2 Institute of Population Health, University of Ottawa, Ottawa, Canada; 3 Department of Information Studies, University of Wales, Aberystwyth, Wales, United Kingdom; 4 C. T. Lamont Primary Health Care Research Centre, Élisabeth Bruyère Research Institute, Ottawa, Canada; 5 Royal college of Physicians and Surgeons of Canada, Ottawa, Canada; 6 Faculty of Medicine, University of Ottawa, Ottawa, Canada; 7 Ottawa Health Research Institute, Ottawa, Canada; 8 Rowan Health Policy Consulting, Ottawa, Canada; University of Otago, New Zealand

## Abstract

**Background:**

The “Just-in-time Information” (JIT) librarian consultation service was designed to provide rapid information to answer primary care clinical questions during patient hours. This study evaluated whether information provided by librarians to answer clinical questions positively impacted time, decision-making, cost savings and satisfaction.

**Methods and Finding:**

A randomized controlled trial (RCT) was conducted between October 2005 and April 2006. A total of 1,889 questions were sent to the service by 88 participants. The object of the randomization was a clinical question. Each participant had clinical questions randomly allocated to both intervention (librarian information) and control (no librarian information) groups. Participants were trained to send clinical questions via a hand-held device. The impact of the information provided by the service (or not provided by the service), additional resources and time required for both groups was assessed using a survey sent 24 hours after a question was submitted. The average time for JIT librarians to respond to all questions was 13.68 minutes/question (95% CI, 13.38 to 13.98). The average time for participants to respond their control questions was 20.29 minutes/question (95% CI, 18.72 to 21.86). Using an impact assessment scale rating cognitive impact, participants rated 62.9% of information provided to intervention group questions as having a highly positive cognitive impact. They rated 14.8% of their own answers to control question as having a highly positive cognitive impact, 44.9% has having a negative cognitive impact, and 24.8% with no cognitive impact at all. In an exit survey measuring satisfaction, 86% (62/72 responses) of participants scored the service as having a positive impact on care and 72% (52/72) indicated that they would use the service frequently if it were continued.

**Conclusions:**

In this study, providing timely information to clinical questions had a highly positive impact on decision-making and a high approval rating from participants. Using a librarian to respond to clinical questions may allow primary care professionals to have more time in their day, thus potentially increasing patient access to care. Such services may reduce costs through decreasing the need for referrals, further tests, and other courses of action.

**Trial Registration:**

Controlled-Trials.com ISRCTN96823810

## Introduction

Delivery of primary care is the foundation of modern effective healthcare systems [1] . Countries that have given priority to and invested in the primary care component of their healthcare systems have the best healthcare outcomes, provide the most equitable care, and are the least expensive [2]. Yet, faced with a rapidly expanding knowledge base, primary care professionals are challenged to remain up-to-date with their professions. Primary care professionals regularly identify answers to some problems as they arise. However, no matter how adept they are, information retrieval takes time [3].

The idea of a question and answering service in primary care has been previously studied both within and outside the library context [4]–[8]. Until recently, the United Kingdom's National Library for Health also piloted a Clinical Question and Answer service. However, the design and evaluation methods that are used in this project are unique and combine information sciences and health services research. The time factor is very unique. This is the only service that we know of that provides primary care clinicians with answer to their clinical questions in under twenty minutes. This short response time meant that the provider could ask most patients to wait for them to receive an answer.

We examined whether we could assist primary care providers in their decision-making by rapidly providing information in response to the clinical questions raised while seeing patients. We hypothesized that the service would save time and reduce the number of questions that were raised but not answered due to time constraints. A recent study reinforced that collaboration with a librarian increased health professionals' willingness to seek information [9]. We therefore felt that librarians (individuals with Master's level training from an American Library Association accredited program) were the best professionals with whom to collaborate. Our research question asked if a librarian consultation service could quickly provide information to help primary care professionals answer their clinical questions during clinic hours.

## Methods

The protocol for this trial and supporting CONSORT checklist are available as supporting information; see Checklist S1 and Protocol S1.

### Participants

Our service targeted primary care providers in Family Health Networks (FHNs) and Family Health Groups (FHGs), two recently introduced models for primary care service delivery in Ontario.

### Design and randomization

We designed a randomized controlled trial (RCT) to evaluate our service using the information provided by a librarian in response to a clinical question (intervention); the RCT was conducted between October 2005 and April 2006 with ethical approval from the Ottawa Hospital Research Ethics Committee. The unit of allocation for RCT randomization was the clinical question. A computer-generated randomization list was subsequently created; an independent company which managed the project's data ensured adequate allocation concealment.

We used a stratified randomization scheme where the strata is the physician and the question is the element that is being randomized. An unequal 3∶1 randomization ratio was used. This ratio was used because it has been suggested that allocating more participants to the intervention group will permit greater experience of a new treatment [10], [11]; in this study, this meant allow more questions to be answered by a librarian.

A random number generator allocated questions between the intervention and control groups. A simulation program was run with 1,000 iterations. The standard deviation of ‘time saved’ was unknown. For sample size estimation it was assumed a minimum clinically important difference of one-quarter standard deviation (in minutes) of the intervention and the control times. A sample of 88 physicians each with 22 questions, five of which were controls, had 99% power to distinguish between the intervention and control groups.

The simulation distinguished between the control and intervention question times using a standard t-test. There were 430 control times, and 1,462 intervention times produced by each iteration of the simulation. These were compared using a standard t-test. Thus the unit of randomization and analysis was the ‘time saved’ for a question. One thousand simulations were performed providing 1,000 t-tests. The power was determined by the percent of the time that the null hypothesis (control time equals intervention time) was rejected.

### Just-in-time (JIT) intervention

The “Just-in-time information” (JIT) librarian consultation service was designed to provide a rapid response to clinical questions during patient visit hours. The questions were submitted by the participants and each question was randomly assigned to the intervention (librarian information) or control (no librarian information) group. If the question was randomized to the control group, participants received a message within one minute that their question would not be answered. The librarian still answered the question, but the software blocked the response from being sent to the participant. Thus, they would need to try to answer the question themselves.

Each participant was asked to respond to a survey 24 hours after a question was submitted, regardless of the allocation. The survey included the information in the question that the participant asked and participants averaged two questions per month. None of the participants, investigators, or librarians knew to which group a question would be randomized. Librarians answered all questions, regardless of the group allocation, and never knew which questions were sent or not sent to the participants. The data was anonymous to the investigators and the statistician who conducted analysis. A research assistant kept the information to link the project data to participants. However, at the time of submitting a survey response, participants did know the randomization of their question (as it was clear to them if they received a response or not).

### Training

Before entering the study, the primary care providers received instructions on using the JIT service, including how to: 1) submit questions and receive answers using the hand-held device and/or website; and 2) formulate clinical questions within the project scope. Well-built clinical questions were required to be relevant to the problem, phrased to facilitate rapid information retrieval, and constructed using the following PICO elements: the patient (or population), the intervention or exposures, the comparison when relevant, and the clinical outcome of interest [12]. “In-scope” questions were defined as clinical questions that could be answered by the librarian within 20 minutes or less. Reference questions that required in-depth research or questions that dealt with drug dosing were considered “out-of-scope” and not answered and participants received a message via their hand-held device. No training was provided to participants about how to locate answers to clinical questions which could bias the results of survey responses to control questions over time.

Three librarians and one library co-operative student (2.26 full-time equivalents) were trained to use evidence-based medicine techniques (including clinical question interpretation, advanced literature searching skills, and critical appraisal) and how to deliver uniform and consistent information.

### Procedures

The design included a “run-in” period of one year prior to randomization to ensure that any service processes that might influence the conduct of the trial were identified and resolved as well as to allow the participants to become used to the service before being exposed to interruptions (control questions). At the beginning of this period, librarians were located in some of the larger clinical practices in order to champion the service. The service allowed participants to submit questions electronically and receive information relevant to their clinical questions. The rapid delivery of the information meant the participant could have a patient wait and apply the information provided before the end of their visit. When a question was received, a librarian searched the literature to locate relevant information. The information and the appropriate citation details were returned to the enquiring participant's hand-held device. A hand-held device (BlackBerry™) was chosen because of its portability in any clinical setting. The BlackBerry™ was programmed to either ring or vibrate when the information was received. All information was stored in a searchable database. Participants also had the option of reviewing their questions and answers via a website. Project librarians followed an evidence-based process to locate one high-quality information citation to assist participants in answering their questions. If there was conflicting information, both sources were sent to the participant. Project librarians had access to the medical literature through the University of Ottawa Library Network.

### Outcomes

We investigated if there were positive impacts for the primary care providers in terms of time, decision-making, cost savings and satisfaction. The time (t_1_) taken to respond to questions and cost savings (time to locate information, workload, and additional healthcare resource use) were the primary outcomes. A measurement of self-reported impact of information received from the JIT service was a secondary outcome. The time (t_2_) and date were electronically stamped when the question was received and the information was sent to the participant. The total librarian time reflected the difference between these two times (t_1_, t_2_). To address other outcomes, a three-part impact assessment survey was sent to participants 24 hours after each time they submitted a question. Part one assessed the perceived cognitive impact of the information provided in response to the clinical questions using an impact assessment scale that evaluated cognitive impact based on the work of Pluye et al [13]. The impact assessment scale included ten categories ranging from high positive impact (enhanced clinical decision-making, learning something new, updating knowledge or recalling something forgotten), moderate positive impact (reassurance or confirmation), no impact to negative impact (too much information, too little information, disagreement with information or potentially harmful information). Participants were asked to select one category that best reflected the information that the JIT service provided (or that they obtained on their own). Part two asked if additional resources were used to address their questions. Part three assessed how much time (recorded in minutes) their response to Part two required. At the end of the study, a satisfaction survey was delivered using a seven-item instrument with mostly closed-ended Likert-scale items.

Clinical questions were categorized using the four main evidence-based methodology categories (diagnosis, therapy, etiology, and prognosis), plus an “other” category [14]. As well, a scale to evaluate complexity was developed (see Table 1). We categorized complexity from 1 (a simple question with no modifiers) to 4 (a question with many modifiers). Only five questions were excluded due to being “out-of-scope”.

**Table 1:** Clinical question levels of complexity.

Level 1	One component for each PICO element
Level 2	One modifier in one component of PICO
Level 3	than one modified in one or more components of PICO
Level 4	Hybrid questions (more than one type of question)

At the end of the study, a general linear regression model was run twice, once with participant time and then with librarian time as the dependent variables. The former model had both groups (control, intervention) and librarian time as the explanatory variables, while the latter had only group (control, intervention) as the explanatory variable.

## Results

**Figure 1:**
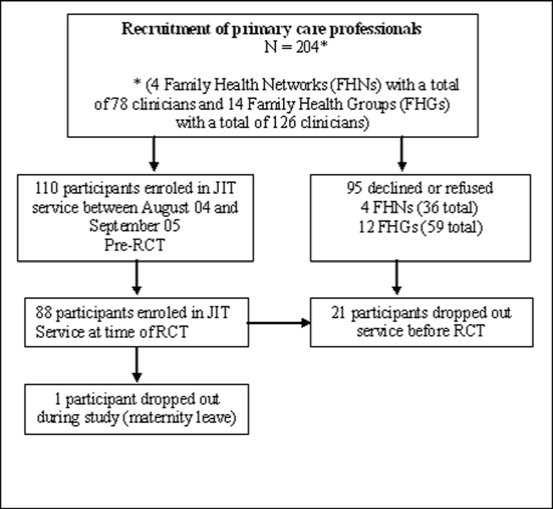
Just-in-time recruitment process.

Four FHNs and 14 FHGs involving 205 primary care providers were identified for potential recruitment (Figure 1). Recruitment occurred in two rounds with a letter and an information sheet provided to potential participants. A follow-up letter was faxed or mailed to those who did not initially respond. Sites were purposefully recruited based on location to simplify librarian travel and training demands. A total of 110 individuals signed consent forms; 21 of these individuals withdrew from participation before randomization, leaving a final group of 88 individuals who participated in the RCT. One physician dropped out the trial early due to a maternity leave. Their characteristics are shown in Table 2. They were mostly physicians (93.2%; n = 82), with a small number of nurse practitioners (4.5%; 4), residents (1.1%; n = 1), and nurses (1.1%; n = 1). There was a similar percentage of males (51.1%; n = 45) and females (48.9%; n = 43). The most frequent age category was 40–49 years (41%; n = 36). The demographics for those who withdrew were similar to included study participants.

**Table 2:** Baseline characteristics of participants.

Type of Primary Care Professional	Frequency (percentage)
Physician	82 (93.2)
Resident	1 (1.1)
Nurse Practitioner	4 (4.5)
Nurse	1 (1.1)
*Total*	88 (100)
**Age Range**	
Under 30	1 (1.1)
30–39	20 (22.7)
40–49	36 (40.9)
50–59	22 (25)
60+	8 (9.1)
No response	1 (1.1)
*Total*	88 (100)
**Years since graduating from medical school**	
In Residency	1(1.1)
5 or less	5 (5.7)
6–10	14 (15.9)
11–15	11 (12.5)
16–20	14 (15.9)
20+	42 (47.7)
*Total*	88 (100)

A total of 1,889 questions were submitted to the service; 472 were randomized to the control group (25%) and 1417 were randomized to the intervention group (75%). The types of clinical questions asked by both groups were similar, as was the time for JIT librarians to answer them (see Table 3). While most questions related to therapy, the questions covered a wide range of patient-related issues (see Table 3). The majority of questions for both groups had a complexity level of 1 (85.0% - intervention; 86.9% - control) and similar times to answer. In both groups, the time to answer questions increased with the level of complexity.

**Table 3:** Types of clinical questions.

Type of clinical question	Control (n = 472/1889)	JIT^1^ time	Intervention (n = 1417/1889)	JIT^1^ time
	Frequency (percentage)	Mean	Frequency (percentage)	
Diagnosis	81 (17.2)	14.5656	253 (17.9)	13.9491
Etiology	99 (21.0)	13.1418	270 (19.1)	14.0780
Other	39 (8.3)	14.4684	104 (7.3)	15.1654
Prevention	67 (14.2)	12.0823	194 (13.7)	13.4092
Prognosis	9 (1.9)	13.1981	36 (2.5)	12.7074
Therapy	177 (37.5)	13.3623	555 (38.2)	13.5038
Out of scope	0		5 (0.4)	8.2400
Total	472 (100)	13.4293	1417 (100)	13.7632

**1 = Just-in-time**.

The primary outcome was time to receive a response, whether time for JIT librarians to locate information to provide a response to a question, or a participant's time to search for the information. The average time for JIT librarians to provide a response all questions was 13.68 minutes/question (95% CI, 13.38 to 13.98) and the average time for participants to respond to a control questions was 20.29 minutes/question (95% CI, 18.72 to 21.86). The average salary cost for a project librarian to respond to a question was approximately $7.15 (based on 15 minutes). The average salary cost for a FGH or FHN physician to respond to question in 15 minutes ranges from $20.75 to $27.69 [15].

The cognitive impact of the provided information on participants' decision-making is reported in Table 4. Of the responses provided to intervention questions, participants rated 63% as having a highly positive impact, 17% as having a moderately positive impact, 7.8% as having no impact, and 7.7% as having a negative impact. Of the responses provided to control questions, participants rated 14.9% as having a highly positive impact, 5.9% as having a moderately positive impact, 24.8% as having no impact, and 44.9% as having a negative impact on decision-making. Participants only attempted to locate answers to 40.5% of control questions themselves. Other actions that they took to answer control questions included: taking no further action (25.4%), asking a practice colleague (6.1%), speaking by phone with another physician (3.8%), having the patient return (1.1%), arranging for a referral (3.0%), or using other actions (9.5%), including sending the patient for a diagnostic test. Participants recorded if they sought additional resources for intervention questions; 59.6% of questions did not require additional resources. For 17% of control questions, participants used additional resources.

**Table 4:** Impact assessment scores.

Impact Assessment scale	Control (n = 472/1889)		Intervention (n = 1417/1889)	
	Frequency	Percent	Frequency	Percent
No answer	45	9.5	62	4.4
***High positive impact***				
1. Practice Improvement: My clinical decision-making was enhanced.	24	5.1	285	20.1
2. Learning: I learned something new or updated my knowledge.	41	8.7	528	37.3
3. Recall: I recalled something I had forgotten.	5	1.1	79	5.6
***High positive impact sub-total***	*70*	*14.9*	*892*	*63*
***Moderate positive impact***				
4. Reassurance: I was more confident.	11	2.3	114	8.0
5. Confirmation: The information confirmed I was doing the right thing.	17	3.6	128	9.0
***Moderate positive impact sub-total***	*28*	*5.9*	*242*	*17*
**No impact**				
6. The information had no impact.	117	24.8	111	7.8
***Negative impact***				
7. There was too much information.	6	1.3	23	1.6
8. There was too little or no information.	205	43.4	80	5.6
9. I disagree with the information.	1	0.2	7	0.5
10. I think the information is potentially harmful.	0	0	0	0
***Negative impact sub-total***	*212*	*44.9*	*110*	*7.7*
**Totals**	472	100.0	1417	100.0

Participants only attempted to locate answers to 40.5% of control questions themselves. Other actions that they took to answer control questions included: taking no further action (25.4%), asking a practice colleague (6.1%), speaking by phone with another physician (3.8%), having the patient return (1.1%), arranging for a referral (3.0%), or using other actions (9.5%), including sending the patient for a diagnostic test. Participants recorded if they sought additional resources for intervention questions; 59.6% of questions did not require additional resources. For 17% of control questions, participants used additional resources.

Most participants (81%; n = 72/88) responded to the exit satisfaction survey. The majority of them rated their level of satisfaction with the service as having a positive impact (86%; n = 62) on the care they provided to their patients and 83% assessed the service as providing relevant information to their questions in an appropriate time frame. Most participants (72%; n = 52) would consider using a similar service, while a small number (33%; n = 24) were willing to pay for such a service. Most participants (82%; n = 59) preferred this service to be delivered by a hand-held device or web interface. A qualitative analysis of open-ended questions from this survey also showed that many participants noted that the service saved them time, improved their access to information, and supported their clinical decision-making with patients. Some participants related improved decision-making to the currency and quickness of the information.

## Discussion

Our study has some limitations. We did not follow-up with participants to determine specifically how they used the time that was saved by the service. We did not examine why the participants felt their information had little or no impact or had potentially negative effects on decision-making. Participants were blinded to randomization statues when submitting question. However, they did know whether or not they received a response and thus were not blinded to randomization status when submitting a survey response.

This RCT indicated that JIT librarians answered clinical questions more quickly than primary care providers. The average time (<15 minutes) was less than our estimated time of twenty minutes used when promoting the project. While participant time (to answer a control question) was just over 20 minutes, this was only for the 40% of questions where the participant chose to try to answer the question. This showed that without the assistance of the service, many clinical questions went unanswered during our study. This finding is similar to a previous study by Ely et al., who reported that physicians did not seek answers to many of their questions (55%), often suspecting a lack of usable information [16]. A recent study compared the effectiveness and costs of providing information for patient care via librarian-mediated searching versus information skills training for health professionals and found that both were similar [17]. Our results also suggest that it is less costly for librarians to locate relevant information than primary care providers. Additional costs to our service included development and support of customized software, a part-time coordinator, and laptops.

Our service decreased the use of consultations with other practice physicians, return patient visits, referrals, and other actions in the control group; reductions in these areas decrease costs. We were unable to determine if the time saved by a librarian was spent in additional primary care, used to see other patients, or allowed the primary care professional to work fewer hours.

Our study shows a unique application of RCT design by combining librarianship with health services research. Using a question as the unit of randomization is unusual. Rather than an individual being randomized, it was a clinical question. Another innovative study component randomized participants to answer clinical questions to see if there were differences between using specific resources versus usual information resources [18].

Another finding in our study was the highly positive impact rating (62.9%) on participants' decision-making abilities based on information provided in response to clinical questions. When participants were left to locate answers to questions themselves, 44.9% of the answers had a negative impact on decision-making and 24.8% had no impact at all. We interpret this to indicate that using a librarian to answer clinical questions provides a large benefit and assistance to the decision-making of our participants. In addition, we conclude that the time participants spent responding to their own clinical questions was not productive or effective for decision-making and thus not an efficient use of their time. Our study quantifies the time required to respond to clinical questions, and builds on the results of two previous studies: the Rochester Study demonstrated that information provision could save physicians time and change their decision-making [19], while a subsequent study from the United Kingdom validated these results [20].

Sir Muir Gray has highlighted the need for access to clean, clear knowledge for health professionals but noted that Canadians do not have a coordinated national approach to a library service [21]. Our results may be generalizable to other primary care populations and suggest that having a JIT librarian consultation service may improve the efficiency and quality of the healthcare system. However, the generalizability of finding should be interpreted with caution due to low enrolment.

Further, the consultation service could likely be provided at an acceptable cost from the perspective of patients and governments. While governments may not experience a direct financial benefit from having the librarian service available to primary care providers, they will see savings by way of reduced visits to specialists, fewer tests, and fewer follow-up appointments.

We have demonstrated that the use of a librarian consultation service saved time for the participants and significantly improved their access to information for patient care decision-making. Further, we found that the information provided by the service had a higher impact than if the clinician searched for information themselves.

## Supporting Information

Protocol S1 Trial Protocol
(0.07 MB DOC)

Checklist S1 CONSORT Checklist
(0.07 MB DOC)
