# Supplementary material for: Just-in-Time Information Improved Decision-Making in Primary Care: A Randomized Controlled Trial
Source: PLoS One. 2008 Nov 21;3(11):e3785. doi: 10.1371/journal.pone.0003785 (PMC2583045; doi:10.1371/journal.pone.0003785)
Supplement: Protocol S1 — Trial Protocol (0.07 MB DOC) [file pone.0003785.s001.doc]

| **'Just-in-time information' librarian support at the point of service delivery for family health networks** | |
| --- | --- |
|  | |
| **ISRCTN** | ISRCTN96823810 |
|  | |
| **Title of trial/grant title** | 'Just-in-time information' librarian support at the point of service delivery for family health networks |
|  | |
| **Acronym** | JIT |
|  | |
| **Serial number at source** | G03-02920 |
|  | |
| **Study hypothesis** | The 'Just-in-time information' (JIT) project is designed to test whether or not a librarian consultation service can have a positive impact in Family Health Networks (FHNs) in terms of cost-effectiveness (saving time, workload issues) and improved access to information. |
|  | |
| **Ethics approval** | Ottawa Health Research Institute, approved on 12 May 2004. Ref: 2004298 |
|  | |
| **Study design** | Randomised controlled trial |
|  | |
| **Countries of trial** | Canada |
|  | |
| **Disease/condition/study domain** | Clinical questions arising in the primary care setting. |
|  | |
| **Participants - inclusion criteria** | Health professionals working in family health networks in Ontario, Canada. |
|  | |
| **Participants - exclusion criteria** | Health professionals not working in family health networks. |
|  | |
| **Anticipated start date** | 01/10/2005 |
|  | |
| **Anticipated end date** | 27/04/2006 |
|  | |
| **Target number of participants** | 80 |
|  | |
| **Interventions** | Clinicians were trained by information specialists (medical librarians) to use a hand-held device to send clinical questions that arise during patient visits to the librarians. All questions were answered by blinded librarians. The answers to questions that had been randomised to intervention were returned to the clinicians who sent the questions. The answers to questions that had been randomised to control were not returned to the clinicians and therefore they had to find the answers themselves. The impact of answers to questions randomised to intervention on clinical decision-making was rated by the clinicians. |
|  | |
| **Primary outcome measure(s)** | 1. Time to answer questions  2. Cost saving |
|  | |
| **Secondary outcome measure(s)** | Assessment of the impact of answers to questions randomised to intervention on clinical decision-making using a 10 point scale. |
|  | |
| **Sources of funding** | Primary Health Care Transition Fund, Ontario Ministry of Health and Long-Term Care (Canada) |
|  | |
| **Contact name** | **Ms  Jessie   McGowan** |
| Address | Institute of Population Health, Room 206  University of Ottawa  1 Stewart Street |
| City/town | Ottawa |
| Zip/Postcode | K1N 6N5 |
| Country | Canada |
| Tel | +1 613 562 5800 x2359 |
| Email | jmcgowan@uottawa.ca |
|  | |
| **Sponsor** | Primary Health Care Transition Fund Unit (Canada) |
| Address | Primary Health Care Transition Fund Unit  Ministry of Health and Long-Term Care  5700 Yonge Street, 3rd Floor  North York, ON |
| City/town | Toronto |
| Zip/Postcode | M2M 4K5 |
| Country | Canada |
| Tel | +1 416 327 8997 |
| Email | allen.paul@moh.gov.on.ca |
|  | |
| **Date applied** | 08/03/2007 |
| **Last edited** | 18/04/2007 |
| **Date ISRCTN assigned** | 18/04/2007 |
|  | |
